# DREAM: a database of experimentally supported protein-coding RNAs and drug associations in human cancer

**DOI:** 10.1186/s12943-021-01436-1

**Published:** 2021-11-13

**Authors:** Shupeng Li, Lulu Li, Xiangqi Meng, Penggang Sun, Yi Liu, Yuntang Song, Sijia Zhang, Chuanlu Jiang, Jinquan Cai, Zheng Zhao

**Affiliations:** 1 Department of Neurosurgery, the Second Affiliated Hospital of Harbin Medical University, Neuroscience Institute, Heilongjiang Academy of Medical Sciences, Harbin, 150086 China; 2 Department of Educational Administration, the Second Affiliated Hospital of Harbin Medical University, Harbin, 150086 China; 3 Department of Microbiology, Tumor and Cell Biology (MTC), Biomedicum, Karolinska Institutet, 171 65 Stockholm, Sweden; 4 Beijing Neurosurgical Institute, Capital Medical University, Beijing, 100070 China

**Keywords:** Drugs, Human cancers, Database, Data visualization

## Abstract

The Drug Response Gene Expression Associated Map, also referred as “DREAM” (http://bio-big-data.cn:8080/DREAM), is a manually curated database of experimentally supported protein-coding RNAs and drugs associations in human cancers. The current version of the DREAM documents 3048 entries about scientific literatures supported drug sensitivity or drug intervention related protein-coding RNAs from PubMed database and 195 high-throughput microarray data about drug sensitivity or drug intervention related protein-coding RNAs data from GEO database. Each entry in DREAM database contains detailed information on protein-coding RNA, drug, cancer, and other information including title, PubMed ID, journal, publish time. The DREAM database also provides some data visualization and online analysis services such as volcano plot, GO/KEGG enrichment function analysis, and novel drug discovery analysis. We hope the DREAM database should serve as a valuable resource for clinical practice and basic research, which could help researchers better understand the effects of protein-coding RNAs on drug response in human cancers.

## Background

Cancer incidence and mortality increase rapidly, making cancer the major cause of death worldwide [1]. Although there are different cancer treatments, drug treatment still remains the most common method of cancer healing [2]. Unfortunately, patients initially have a favorable response to drugs, but majority of them will eventually relapse and become drug resistant [3]. So, studying the different expressions of the drug targeting genes and drug-resistant genes are extremely important to predicting novel drug targets and reversing drug resistance. Gene expression is the synthesis of functional gene products such as RNA and protein using the information provided by deoxyribonucleic acid [4]. Over the past decades, the ‘central dogma’ of molecular biology has been used to describe protein-coding RNAs as the information-carrying intermediate in protein synthesis which control various biological functions [5]. In addition, several studies suggest that the dysfunction of the protein-coding RNAs can often influence cancer development [6]. Mounting evidences have proven that drugs can target protein-coding RNAs and protein-coding RNAs can also play critical roles in drug resistance [7, 8]. Hence, protein-coding RNAs can be valuable to pinpoint candidate biomarkers, therapeutic targets, and gene signatures for drug resistance.

With the rapid development and continuous decreasing costs of experimental technology such as high-throughput microarray technology, the number of scientific literatures and microarray data are obviously increasing. The overwhelming amount of cancer related data are increasingly complex for basic researchers to perform data mining, integration, and analysis. These large number of the scientific literatures and high-throughput microarray data are an obstacle to characterizing protein-coding RNAs functions in cancer drugs from a global view.

To bridge this gap, we developed the “Drug Response Gene Expression Associated Map” database, also referred as “DREAM” (http://bio-big-data.cn:8080/DREAM), a comprehensive protein-coding RNAs and drugs associations database from scientific literatures and high-throughput data, which aims to provide a resource for efficient browsing and analysis of drugs and protein-coding RNAs molecules associations directly.

### Database content and functions

In the current version of DREAM database, all the protein-coding RNAs are divided into two categories: drug intervention related protein-coding RNAs, representing protein-coding RNAs that act as drug targets, and drug sensitivity related protein-coding RNAs, representing protein-coding RNAs that are implicated in drug resistance. The current database contains 3048 literatures supported drug sensitivity or drug intervention related protein-coding RNAs entries and 195 drug sensitivity or drug intervention related protein-coding RNAs microarray entries. Each entry in DREAM contains detailed information on protein-coding RNA, drug, cancer, and other information including title, PubMed ID, journal, publish time. All the entries totally embrace 1560 protein-coding RNAs, 138 kinds of drugs, and 35 human diseases.

**Fig. 1:**
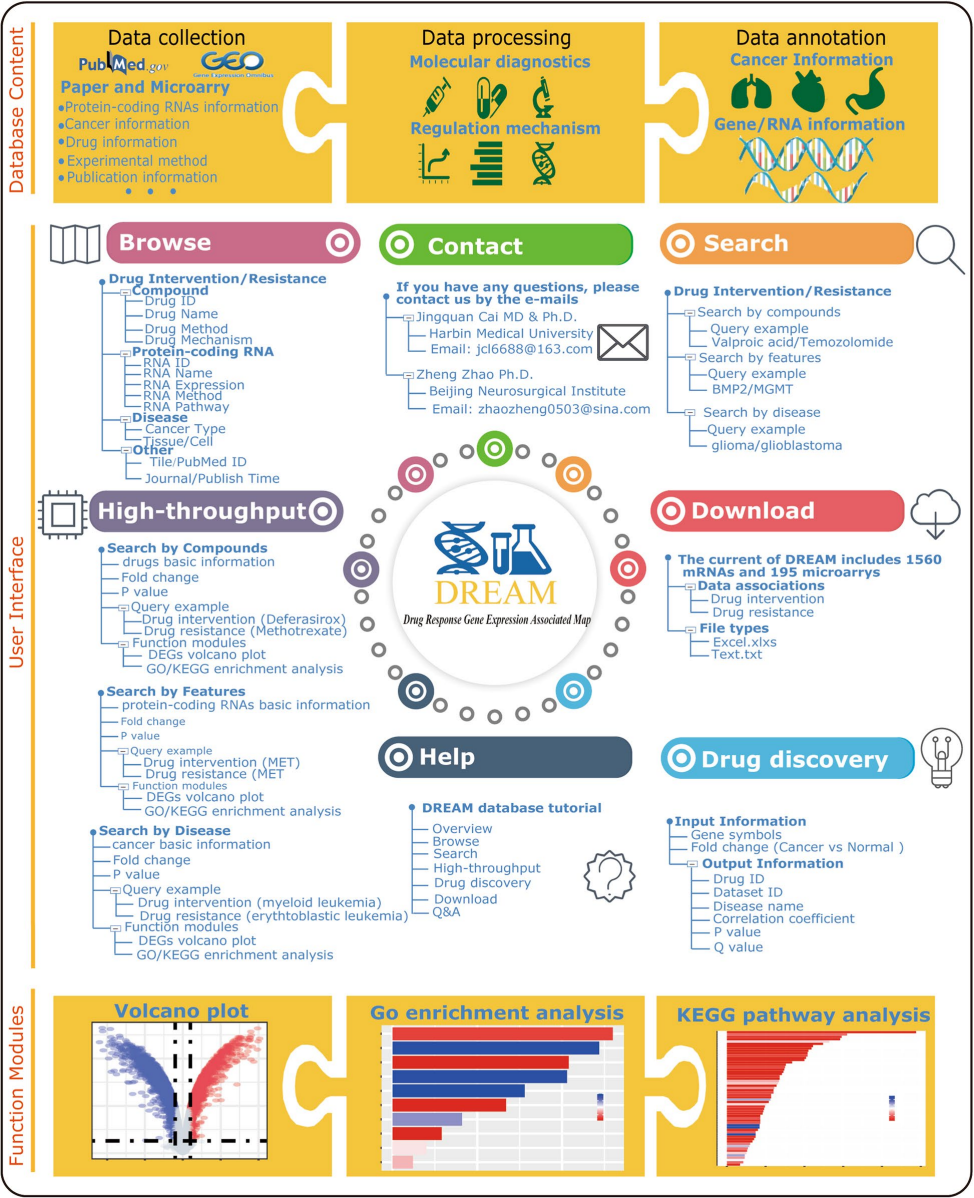
The workflow of the construction of DREAM database

To construct a high-quality database of protein-coding RNAs, all the literatures related to protein-coding RNAs and drugs are manually extracted from publications as previous studies [9] (Fig. 1). We firstly performed an extensive literatures query of PubMed database using a list of keywords, such as ‘protein-coding RNA’ and ‘drug’ or ‘drug target’ or ‘drug sensitivity’ or ‘drug resistance’ and ‘cancer’ or ‘tumor’. There are more than 25,000 abstracts after searching. Then, all retrieved literatures were preliminarily reviewed to remove irrelevant articles by reading abstracts. Only entries with high confidence experiments, such as PCR, Western-blot or luciferase reporter assay, and other reliable methods were recruited. At the same time, we also searched the GEO database using the keywords ‘cancer expression’, and ‘drug’. We got more than 21,000 high-throughput data. Then, we filtered all data by reading the description of the gene chips to retain the eligible data. To share information among other authoritative reference databases, we unified the information by certain criteria, including the drug basic information from DrugBank (https://go.drugbank.com/) and PubChem (https://pubchem.ncbi.nlm.nih.gov/), protein-coding RNAs basic information from Ensembl (https://m.ensembl.org/index.html), and cancer basic information from Disease Ontology (https://disease-ontology.org/).

**Fig. 2:**
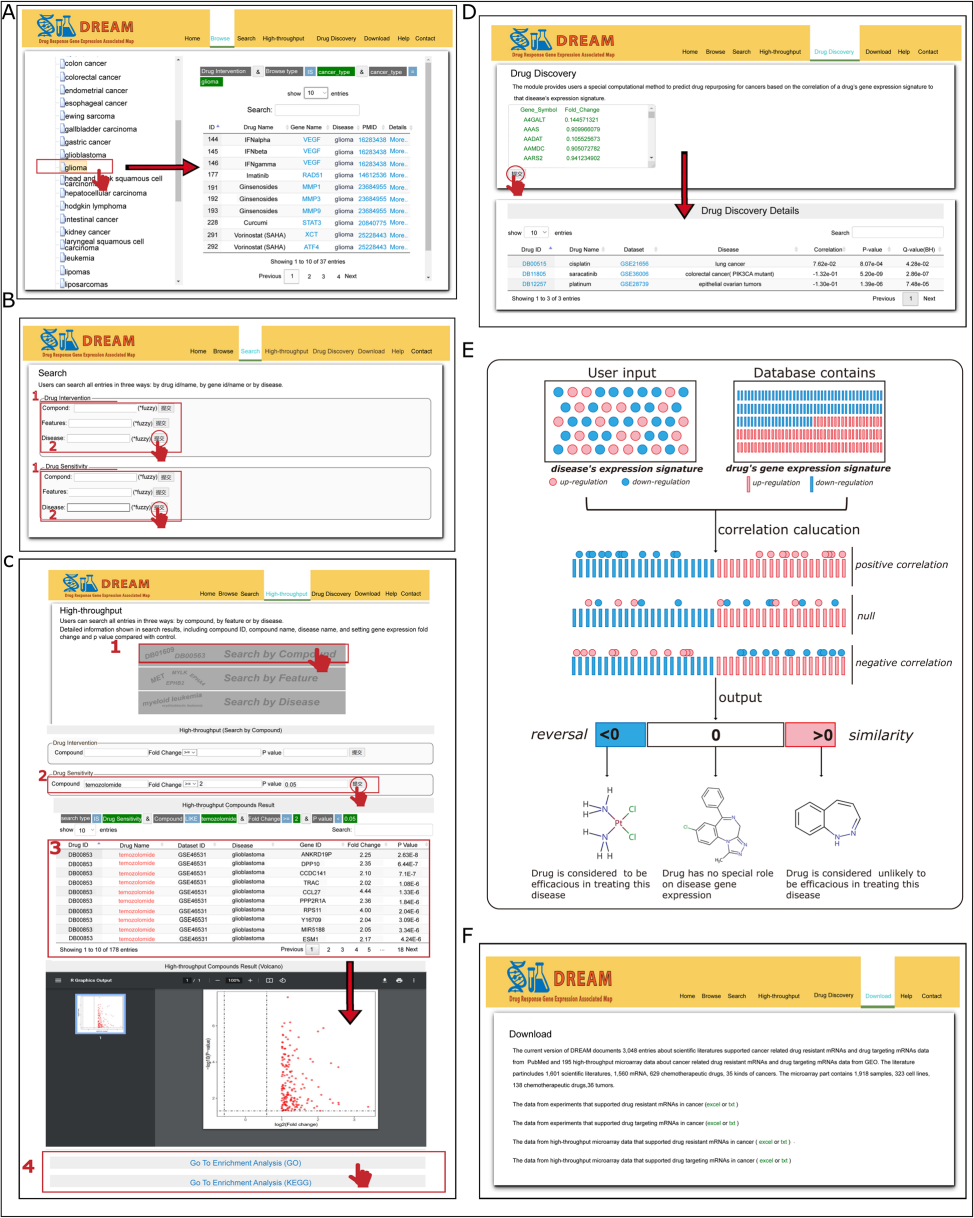
The functions of the DREAM database **A** Browsing function **B** Searching function **C** High-throughput function **D** Drug discovery function **E** The mechanism of drug discovery **F** Download function

DREAM offers a user-friendly web interface to easily browse, search, analysis, and download data. Fig. 1 shows the schematic workflow of DREAM database. (i) In ‘Browse’ page (Fig. 2A), users can browse protein-coding RNAs and cancer drugs association data in DREAM through three ways: by compound name, by gene name, or by disease name. Then, a list of matched entries will be returned. (ii) In ‘Search’ page (Fig. 2B), we permit users to search our database by using protein-coding RNAs names, drug names or DrugBank ID, and disease. DREAM offers fuzzy keyword searching functions, facilitating searches by returning the closest possible matching records. (iii) In ‘High-throughput’ page (Fig. 2C), users can search various cancers’ high-throughput microarray data for drug intervention or drug sensitivity related data. Our database provides user with three different searching methods such as by compound name, by gene name, or by disease name. User can set the statistical significance (*p*-values) and biological significance (fold changes) for different high-throughput microarray data in order to quickly identify the most interesting gene candidates associated with drug targets or drug resistance. The p-values and fold changes are calculated on the basis of case group relative to control group. In “drug intervention” module, the case group is disease cells with drug intervention. The control group is disease cells with placebo intervention such as PBS, DMSO. In “drug sensitivity” module, the case group is drug resistant disease cells and the control group is normal disease cells. In addition, we also provide several functions for user to do further analysis of selected interested results. We implement interactive visualization tools such as volcano plot and add the widely used gene enrichment analysis such as GO annotations and KEGG pathways analysis in this page. (iv) In ‘Drug Discovery’ page (Fig. 2D), the DREAM offers a special computational method to predict drug repurposing for cancers based on the correlation of a drug’s gene expression signature to that disease’s expression signature according to the Kewalin’s method [10]. The raw R code is displayed in the “Help” page of our website. The “drug’s gene expression signature” is extracted from drug-disease related gene expression profiles in DREAM. The “disease’s expression signature” is gene sets with expressed protein-coding RNAs in disease groups relative to healthy control groups. The “disease’s expression signature” demands user upload by themselves and must be composed of gene symbol and fold changes. This method calculates a correlation coefficient between disease’s expression signature and drug’s gene expression signature. A correlation coefficient below zero indicating a complete ‘drug-disease’ reversal effect and above zero indicating a perfect ‘drug-disease’ similar effect. A novel drug indication for a particular disease of interest is identified based on the extent to which drug’s gene expression signature is a ‘reversal’ of disease’s expression signature. The mechanism of the drug discovery was presented in Fig. 2E. In this page, user needs to upload the disease’s expression signature including gene symbols and fold changes (relative to a healthy control). Then, our database would match and calculate the correlation coefficients based on the database containing drug’s gene expression signature. Lastly, the database would return the results including correlation coefficient, drugs, cancers, and *p*-value. The correlation coefficient below zero was considered as significant results which meant the selected drug could reverse the cancer gene expression. For example, by using ‘Drug Discovery’ function we found that Saracatinib, a drug often given to patients with colorectal cancer [11], may also benefit glioma patients. (iv) In ‘Download’ page (Fig. 2F), our database provides two formats of the downloadable file in text and excel formats, respectively. Besides the above content, the web proved some query examples, it also helps users better understand how to use DREAM.

In conclusion, compared with other databases, the DREAM includes three distinctive features: (i) as the first database that provide the associations between the protein-coding RNAs and drugs in human cancer. (ii) merging the literatures data and high-throughput data in a single database. (iii) offering a special computational method to predict drug repurposing for cancers based on the correlation of a drug’s gene expression signature to that disease’s expression signature. In the future, we will continue to collect the protein-coding RNAs and update the database every 3 months. We will improve our database by adding other analysis tools. Anyway, more and more biological data and functional web-based tools will be integrated into DREAM database, which will provide a reliable database platform for a wide range of scientific researchers.

## Data Availability

All data obtained and/or analyzed in this study were available from the DREAM (http://bio-big-data.cn:8080/DREAM).

## Abbreviations

GEO: Gene Expression Omnibus
KEGG: Kyoto Encyclopedia of Genes and Genomes
PCR: Real time polymerase chain reaction
